# Hypokalemic periodic paralysis in a teenage boy after an intense period of exercise: A rare case report

**DOI:** 10.1002/ccr3.8201

**Published:** 2023-11-14

**Authors:** Sahar Noor, Abdul Jamil Rasooly, Sultan Mahmood Alikozai, Tooryalai Jalalzai, Ahmed Maseh Haidary, Najla Nasir, Sarah Noor, Masooma Farooqi, Husna Mansoori

**Affiliations:** ^1^ Department of Pediatrics Medicine French Medical Institute for Mothers and Children (FMIC) Kabul Afghanistan; ^2^ Department of Pathology French Medical Institute for Mothers and Children (FMIC) Kabul Afghanistan; ^3^ Department of Medicine Rabia Balkhi Hospital Kabul Afghanistan; ^4^ Department of Hemato‐Oncology Ali Abad Teaching Hospital Kabul Afghanistan; ^5^ Department of Cardiology French Medical Institute for Mothers and Children (FMIC) Kabul Afghanistan; ^6^ High School Graduate of Tuzluçağır Ankara Turkey

**Keywords:** hypokalemia, muscle weakness, neuromuscular, periodic paralysis

## Abstract

**Key Clinical Messages:**

- Diagnosis of rare even can be missed due to less familiarity with the disorder.
- In patients with muscle weakness, infectious causes are prioritized.
- Electrolyte profile not only identifies the problem, but also prevents unnecessary workup.

**Abstract:**

In underdeveloped countries, diagnosis of rare disorders is usually delayed due to less familiarity of the clinicians to such disorders. As a result, infectious and inflammatory causes for an ailment are prioritized as compared to non‐infectious etiologies. Hypokalemic periodic paralysis (PP) is a rare disorder, characterized by episodic muscle weakness that can rarely be associated with life‐threatening cardiac arrhythmia. A teenage Afghan boy presented to the emergency department with an acute flaccid paralysis, that started 1 h after intense exercise The weakness involved both, the upper and lower extremities. Laboratory investigations, led to the impression of hypokalemic PP, precipitated by intense exercise. Accordingly, intravenous potassium chloride infusion diluted with normal saline led to the complete resolution of paralysis as well as correction of electrocardiographic changes. The list of differential diagnosis for flaccid muscle paralysis is wide, which generally requires a extensive investigations, but in hypokalemic PP, a cardinal electrolytes profile can lead towards early diagnosis. High degree of clinical suspicion with appropriate history taking and physical examination helps with the immediate identification and management of this disorder.

## 1 BACKGROUND

The most prominent clinical manifestations of hypokalemia are neuromuscular, although other systems, such as the cardiovascular as well as gastrointestinal systems may also be affected. Hypokalemic periodic paralysis (HPP) is a rare disorder which is clinically characterized by episodic muscle weakness and occasionally life‐threatening arrhythmia, frequently following strenuous physical activity.^1^, ^2^ Very severe hypokalemia can cause paralysis involving respiratory, bulbar, and cranial musculature.^3^

Although, total potassium stores in the body are sufficient, the migration of potassium into muscle cells causes serum potassium to fall, which renders muscles electrically inactive. The precise mechanism of potassium translocation is unknown, but it has been postulated to be an anomaly in the muscle membrane channel proteins. Recent electrophysiological studies have suggested that the fundamental defect in HPP may be related to an increase in muscle membrane sodium permeability.^4^, ^5^ Genetic linkage data have also suggested that the defect in HPP may be within a dihydropteridine‐binding, voltage‐sensitive, skeletal muscle calcium channel.^6^

HPP is the most common cause of periodic paralysis (PP) with an estimated prevalence of 1 in 100,000.^7^ This disorder is more commonly expressed in males than in females.^8^ It can be primary with autosomal dominant inheritance or acquired.^9^ Heavy exercise, carbohydrate‐rich evening meals, pregnancy, gastrointestinal losses due to vomiting, diarrhea or combination of both and excessive menstruation are usually the triggering factors. Episodic weakness occurs due to drop in the concentration of extracellular potassium ion. The final pathway is aberrant depolarization that inactivates ion channels leading to muscle fiber in‐excitability.^1^, ^10^, ^11^ Here we present a case of HPP, in which flaccid paralysis was triggered by an episode of strenuous exercise.

## 2 CASE PRESENTATION

A 15‐year‐old teenage male patient with 30 kg body weight presented to emergency department with an acute onset of flaccid paralysis. The weakness was bilateral and involved both proximal and distal muscles of upper and lower limbs, without any associated pain. The weakness developed after one and a half hours session of intense exercise. He had no respiratory difficulty or problem in swallowing and was able to move his face and neck without any difficulty. The patient had joined gym for the first time. He was previously healthy but had a sedentary lifestyle. He denied any recent diarrhea, chest pain, palpitation, shortness of breath, or weight change. He had no significant past medical or surgical history and wasn't on any long‐term medication. He was a student, attending ninth grade of school and had never experienced such an event in the past.

Upon physical exam, he was well looking with stable vital signs. On neurological exam he was having flaccidity of upper and lower limbs, overall muscle power of 1/5. Deep tendon reflexes were also diminished scoring overall 2/4. The sensation was intact. There were no fasciculation or obvious muscle atrophy. All the cranial nerves were intact. Cardiac, respiratory and abdominal examinations were normal.

Routine laboratory examination demonstrated a normal CBC, RFT, thyroid profile, creatine phosphokinase (CPK) and ALT values, shown in Table 1. The serum electrolytes at the time of presentation demonstrated very low potassium (K) of 2.2 mEq/L, with normal sodium (Na) of 145 mEq/L, chloride (Cl) of 113 mmol/L, Bicarbonate (Bic) of 18.5 mEq/L, Calcium (Ca) of 7.6 mEq/L, and magnesium (Mg) of 2.1 mEq/L, also shown in Table 1. The ECG showed flattened T wave and a small U wave. On urine exam was normal with no trace of myoglobin, WBC, or RBC cast. Similarly, the thyroid profile was normal.

**TABLE 1:** Laboratory investigations.

Parameters	Results	Follow‐up results	Normal values
Serum Na	145	148	135–145 mEq/L
Serum K	2.2	4.7	3.5–5.5 mEq/L
Serum Cl	113	111	96–106 mmol/L
Serum Bic	18.5	22	21–28 mEq/L
Serum Ca	7.6	8.1	9–11 mg/dL
Serum Mg	2.1	2	1.5–2.3 mEq/L
Hemoglobin	14	–	13.8–17.2 g/dL in males
White cell count	7.5	–	4.5–11.000/mL
Platelet	408	–	150–450.000/mL
BUN	21	–	6–24 mg/dL
CR	0.7	–	0.7–1.3 mg/dL
ALT	27	–	<45 IU/L
CRP	0.9	–	<1 mg/dL
CPK	100	–	10–120 mcg/L
TSH	3	–	0.4–4 mU/L
T3	5.6	–	3.5 7.8 nmol/L
Free T4	21	–	9–25 pmol/L
Urine Na	–	53	>20 mEq/L
Urine K	–	32	>20 mEq/L

Abbreviations: ALT, alanine‐transaminase; BUN, blood urea nitrogen; CPK, creatine phosphokinase; CR, creatinine; CRP, c‐reactive protein; dL, deciliter; K, potassium; L, liter; mEq, milli‐equivalent; mg, milligram; mL, microliter; Na, sodium; T3, tri‐iodo‐thyronine; T4, Thyroxine tetra‐iodo‐thyroinine; TSH, thyroid stimulating hormone.

With an impression of HPP, precipitated by strenuous exercise, the patient was started on IV potassium chloride (KCL) infusion diluted with normal saline. He responded quickly as the paralysis resolved immediately and ECG changes reverted to normal within first hour of KCL infusion. The repeat potassium level was 2.8 mEq/L. Patient was educated about condition, advised to avoid precipitants, and after observation for more than 48 h in the ward he was discharged with oral KCL tablets along with calcium and vitamin D3 supplements. On follow‐up visit after 3 days, he had no complaints. His electrolytes panel as well as urine sodium and urine potassium were normal, as shown in the Table 1.

## 3 DISCUSSION

Our patient presented with HPP after an hour of strenuous exercise, that resolved with timely administration of potassium. HPP is suspected when an individual present with a sudden attack of flaccid muscle weakness involving proximal muscles with absent, decreased or normal deep tendon reflexes following any of the triggering factors. The sensory system is always intact. The clinical suspicion is further supported if positive family or personal history is present.^10^, ^11^, ^12^ The severity can range from mild, transient weakness to severe disabling flaccid paralysis, resulting in life threatening respiratory failure. Low serum potassium level confirms diagnosis at the time of presentation, but to rule out secondary causes; however, several laboratory tests are required. These include thyroid function test (TSH, T3, and T4) to exclude hyperthyroidism, creatine phospho kinase as well as myoglobin to rule out rhabdomyolysis and ECG to look for changes consistent with hypokalemia, which may show T‐wave flattening, U waves and ST‐segment depression, leading to AV block and subsequent cardiac arrest due to arrhythmia.

Electromyography result is often normal, specifically between the weakness episodes when patient is asymptomatic but it can reveal some abnormalities in some patients. This disorder can be present in patients with thyrotoxicosis, barium poisoning, renal disorders like proximal, and distal renal tubular acidosis,^3^ water intoxication, syndrome of inappropriate antidiuretic hormone (ADH) secreation (SIADH),^13^ nephrotic syndrome,^14^ barter's syndrome,^15^ diuretic phase of acute tubular acidosis,^16^ treatment phase of diabetic ketoacidosis,^17^, ^18^ chlorothiazide‐associated hypokalemia,^19^ hypokalemia following rectosigmoidiscopy,^20^, ^21^ gastro‐intestinal losses due to coeliac disease,^22^ tropical sprue,^23^ acute gastroenteritis,^24^ malabsorption syndromes,^25^ endocrinopathies like primary hyperaldosteronism^26^ and pseudohyperaldosteronism induced by licorice (glycyrrhizic acid) ingestion.^27^

The management of HPP includes both therapeutic agents and avoiding precipitating triggers through dietary as well as lifestyle changes, having small frequent meal for avoiding high carbohydrate load, low sodium diet and avoidance of hyperosmolar state such as dehydration and hyperglycemia, decrease the number of attacks. Pharmacological therapies include oral potassium chloride (20–30 mEq/L, every 15–20 min) for acute attacks and carbonic anhydrase inhibitor diuretics (CAIs), particularly acetazolamide and dichlorphenamide, for prevention of subsequent attacks.^1^, ^3^

The mechanism of action of CAIs in HPP is unclear but it promotes kaliuresis and nonanion gap acidosis by increasing urinary bicarbonate excretion and thus systemic acidosis.^28^, ^29^, ^30^ In addition, it also enhances opening of calcium‐activated K channels^31^ and reduces intracellular sodium accumulation.^32^, ^33^ This all culminates to reduces the susceptibility to PP.

HPP during pregnancy and surgery should be given special consideration, because drugs like acetazolamide and dichlorphenamide are FDA pregnancy category C, as a result, using them is challenging and it is important to consider the risk versus benefit ratio. Similarly, extra caution should be taken during surgery and serum electrolytes, especially K level monitoring during and after surgery should be prioritized.^34^

Prognosis of HPP varies among individuals, in which first attacks are associated with good prognosis but repeated paralytic episodes can also be dangerous, due to cardiac arrhythmia but also due to probable involvement of respiratory muscles and associated aspiration pneumonia. Overall, it is worrisome for patients as well as the relatives because it affects patient's social and professional life. Long‐term use of carbonic anhydrase inhibitors for prevention of subsequent attacks can be harmful, as some patients may also develop myopathy and nephrolithiasis. Therefore judicious use of CAIs is advised whenever indicated.^33^, ^35^, ^36^

## 4 CONCLUSION

Muscle paralysis has a wide differential diagnosis that includes infections, neurologic, metabolic, inflammatory, which generally requires a long list of laboratory investigations. Knowledge of the condition as well as detailed clinical history are indispensable to identify HPP, for which just a cardinal electrolytes profile can be sufficient to stop the search for other causes of hypokalemia.

## AUTHOR CONTRIBUTIONS

**Sahar Noor:** Conceptualization; data curation; investigation; methodology; project administration; resources; validation; writing – original draft; writing – review and editing. **Abdul Jamil Rasooly:** Conceptualization; investigation; supervision; validation; writing – original draft. **Sultan Mahmood Alikozai:** Methodology; visualization; writing – original draft. **Ahmed Maseh Haidary:** Supervision; validation; writing – original draft; writing – review and editing. **Tooryalai Jalalzai:** Supervision; validation. **Najla Nasir:** Supervision; validation; writing – original draft. **Sarah Noor:** Supervision; validation; writing – original draft. **Masooma Farooqi:** Investigation; writing – original draft. **Husna Mansoori:** Conceptualization; validation.

## FUNDING INFORMATION

The authors received no funding for current writing.

## CONFLICT OF INTEREST STATEMENT

The authors declare to have no competing interests.

## ETHICS STATEMENT

Ethical approval was acquired from the hospital's ethical review committee. Informed consent was obtained from the patient's legal guardian (father) for participation in the current case report.

## CONSENT

Written informed consent was obtained from the patient's legal guardian for publication of this case report. A copy of the written consent shall be made available for review by the Editor‐in‐Chief of this journal, upon reasonable request.

## Data Availability

All generated data is included in this article.
